# The neglected continuously emerging Marburg virus disease in Africa: A global public health threat

**DOI:** 10.1002/hsr2.1661

**Published:** 2023-10-29

**Authors:** Devang Srivastava, Lakshmi Venkata Simhachalam Kutikuppala, Pooja Shanker, Rudra Narayan Sahoo, Gurudutta Pattnaik, Rasmita Dash, Venkataramana Kandi, Azaj Ansari, Snehasish Mishra, Dhruv N. Desai, Ranjan K. Mohapatra, Ali A. Rabaan, Md. Kudrat‐E‐Zahan

**Affiliations:** ^1^ Department of General Medicine Kakatiya Medical College Rangam Peta Street Warangal Telangana India; ^2^ Department of General Surgery Dr NTR University of Health Sciences Vijayawada Andhra Pradesh India; ^3^ Department of Microbiology SMS Medical College Gangawal Park, Adarsh Nagar Jaipur Rajastan India; ^4^ School of Pharmaceutical Sciences Siksha‐O‐Anusandhan Deemed‐to‐be‐University Bhubaneswar Odisha India; ^5^ School of Pharmacy and Life Sciences Centurion University of Technology and Management Odisha India; ^6^ Department of Microbiology Prathima Institute of Medical Sciences Karimnagar Telangana India; ^7^ Department of Chemistry Central University of Haryana Mahendergarh Haryana India; ^8^ School of Biotechnology KIIT Deemed‐to‐be University Bhubaneswar Odisha India; ^9^ School of Veterinary Medicine, Ryan Veterinary Hospital University of Pennsylvania Philadelphia Pennsylvania USA; ^10^ Department of Chemistry Government College of Engineering Keonjhar Odisha India; ^11^ Molecular Diagnostic Laboratory Johns Hopkins Aramco Healthcare Dhahran Saudi Arabia; ^12^ Department of Medicine, College of Medicine Alfaisal University Riyadh Saudi Arabia; ^13^ Department of Public Health and Nutrition The University of Haripur Haripur Pakistan; ^14^ Department of Chemistry Rajshahi University Rajshahi Bangladesh

**Keywords:** epidemiology, Marburg virus, mitigation strategy, one‐Health approach, risk assessment, therapeutics, zoonosis

## Abstract

**Background and Aim:**

Severe viral hemorrhagic fever (VHF) is caused by Marburg virus which is a member of the Filoviridae (filovirus) family. Many Marburg virus disease (MVD) outbreaks are reported in five decades. A major notable outbreak with substantial reported cases of infections and deaths was in 2022 in Uganda. The World Health Organisation (WHO) reported MVD outbreak in Ghana in July 2022 following the detection of two probable VHF patients there. Further, the virus was reported from two other African countries, the Equatorial Guinea (February 2023) and Tanzania (March 2023). There have been 35 deaths out of 40 reported cases in Equatorial Guinea, and six of the nine confirmed cases in Tanzania so far.

**Methods:**

Data particularly on the several MVD outbreaks as reported from the African countries were searched on various databases including the Pubmed, Scopus, and Web‐of‐science. Also, the primary data and reports from health agencies like the WHO and the Centers for Disease Control and Prevention CDC) were evaluated and the efficacy reviewed.

**Results:**

Chiroptera in general and bat species like *Rousettus aegyptiacus* and *Hipposideros caffer* in particular are natural reservoirs of the Marburg virus. MVD‐infected nonhuman primate African fruit‐bat and the MVD‐infected humans pose significant risk in human infections. Cross‐border viral transmission and its potential further international ramification concerns raise the risk of its rapid spread and a potential outbreak. Occurrence of MVD is becoming more frequent in Africa with higher case fatality rates. Effective prophylactic and therapeutic interventions to counter this deadly virus are suggested.

**Conclusion:**

In the face of the lack of effective therapeutics and preventives against MVD, supportive care is the only available option which contributes to the growing concern and disease severity. In view of the preventive approaches involving effective surveillance and monitoring system following the “One Health” model is extremely beneficial to ensure a healthy world for all, this article aims at emphasizing several MVD outbreaks, epidemiology, zoonosis of the virus, current treatment strategies, risk assessments, and the mitigation strategies against MVD.

## 1 INTRODUCTION

Marburg virus disease (MVD) was reported in Germany and Serbia as the first case, in 1967.^1^ MVD is viewed as a dangerous and lethal infection since then, mostly in the African subcontinent. Four confirmed MVD cases and three deaths with 75% case fatality ratio (CFR) were reported from Ghana (Ashanti region) last year till July 29, 2022,^2^ the first‐ever declared MVD outbreak there. Recently reported outbreak in Equatorial Guinea and Tanzania of the deadly MVD has sparked an alarm and global fear, especially due to the fact that these nations are still battling the COVID‐19 ongoing pandemic. Equatorial Guinea reported 12 deaths, 17 confirmed, and 23 probable cases, which led to the WHO declaring MVD as an outbreak in February 2023.^3^ Although Equatorial Guinea confirmed MVD cases in Kie Ntem province initially, however, Bata in Litoral province was the most affected district.^2^ All the probable 23 cases died later. Tanzania had recorded eight confirmed cases and five deaths till March 24, 2023.^4^ One probable case also died. All the cases reported there were from Bukoba district of Kagera region. While the origin of the recent Ghana and Equatorial Guinea MVD outbreak is unidentified, the Tanzania outbreak was linked to Goziba Island in Lake Victoria, Tanzania. Nonetheless, these current MVD outbreaks were the first identified and reported from Ghana, Equatorial Guinea and the United Republic of Tanzania.

Known as Marburg hemorrhagic fever (MHF) earlier, MVD is certainly an illness of concern in humans and nonhuman primates that could turn fatal,^1^, ^5^ characterized by hemorrhagic fever with high morbidity and fatality rates (66.7%–75% CFR). Represented by one Ravn virus sp., Marburg virus, like Ebola, belongs to family Filoviridae. The virus is pleomorphic which could be filamentous (892 nm length and 91 nm diameter), circular, or in the configuration of the digit “6”.^6^ Typically they are shepherd's crook shaped, “U” shaped, “6” shaped (like filoviruses) filaments, and could also be coiled, toroidal, or branched. These virions typically are of 80 nm width and may slightly vary in length. They typically have a median length of 795–828 nm as opposed to Ebola virion which has 974–1086 nm median length, although tissue culture revealed the viral particles as 14,000 nm large.^7^ Like all mononega viruses, MVD virions are noninfectious, linear, nonsegmented, single‐stranded RNA genomes with negative polarity, inverse‐complementary 3′ and 5′ termini, no 5′ cap, no polyadenylation, and no covalent protein attachment. Glycoprotein, viral protein 4, viral protein 24, nucleoprotein, viral protein 35 (polymerase cofactor), viral protein 30 (transcription activator) and L (RNA‐dependent RNA polymerase) are the seven proteins encoded by this lipid‐enveloped Marburg virus. MVD virus genome is about 19 kbp long and contains seven genes in 3′‐UTR‐NP‐VP35‐VP40‐GP‐VP30‐VP24‐L‐5′‐UTR sequence.^8^ The cells lacking the Niemann‐Pick C1 (NPC1) survived and seemed immune to Ebola virus at least in lab settings, proving that the virus needed NPC1 for cell entry.^9^ Thus, NPC1 cholesterol transporter protein is assumed to be needed in the infection of Marburg and Ebola viruses.

Ugandan studies identified the bat species *R. aegyptiacus* as the MVD virus reservoir.^6^, ^10^ Direct or indirect contacts with fluids transmitted the disease to humans and others.^11^, ^12^ Due to the associated significant risk to public health, MVD virus is categorised as a Tier‐1 select agent by the Centers for Disease Control and Prevention (CDC) and a high priority category A pathogen by the WHO.^13^ The WHO classifies it as a pathogen in risk group 4 requiring biosafety level 4‐equivalent containment in view of its lethality.^14^ We emphasise on several MVD outbreaks, epidemiology, zoonosis of the virus, current treatment strategies, risk assessments and the mitigation strategies to counter the spread of this fatal disease.

## 2 MATERIALS

This narrative review work started with sourcing of data from published literature available on public domain. Reports on the several MVD outbreaks in African countries were searched from various databases including the Pubmed, Scopus, and Web‐of‐science. The primary data and reports from health agencies like the WHO and CDC were also evaluated and reviewed. A total of 53 articles/data sets were shortlisted out of a total 112 collated after searching, and the references were validated and manually listed. To avoid defocusing from the write‐up, irrelevant or less meaningful articles were excluded. To ensure the accuracy of the facts and figures, they were meticulously extracted from the shortlisted articles and reviewed.

## 3 RESULTS AND DISCUSSION

### 3.1 MVD Outbreaks to date

**Figure 1:**
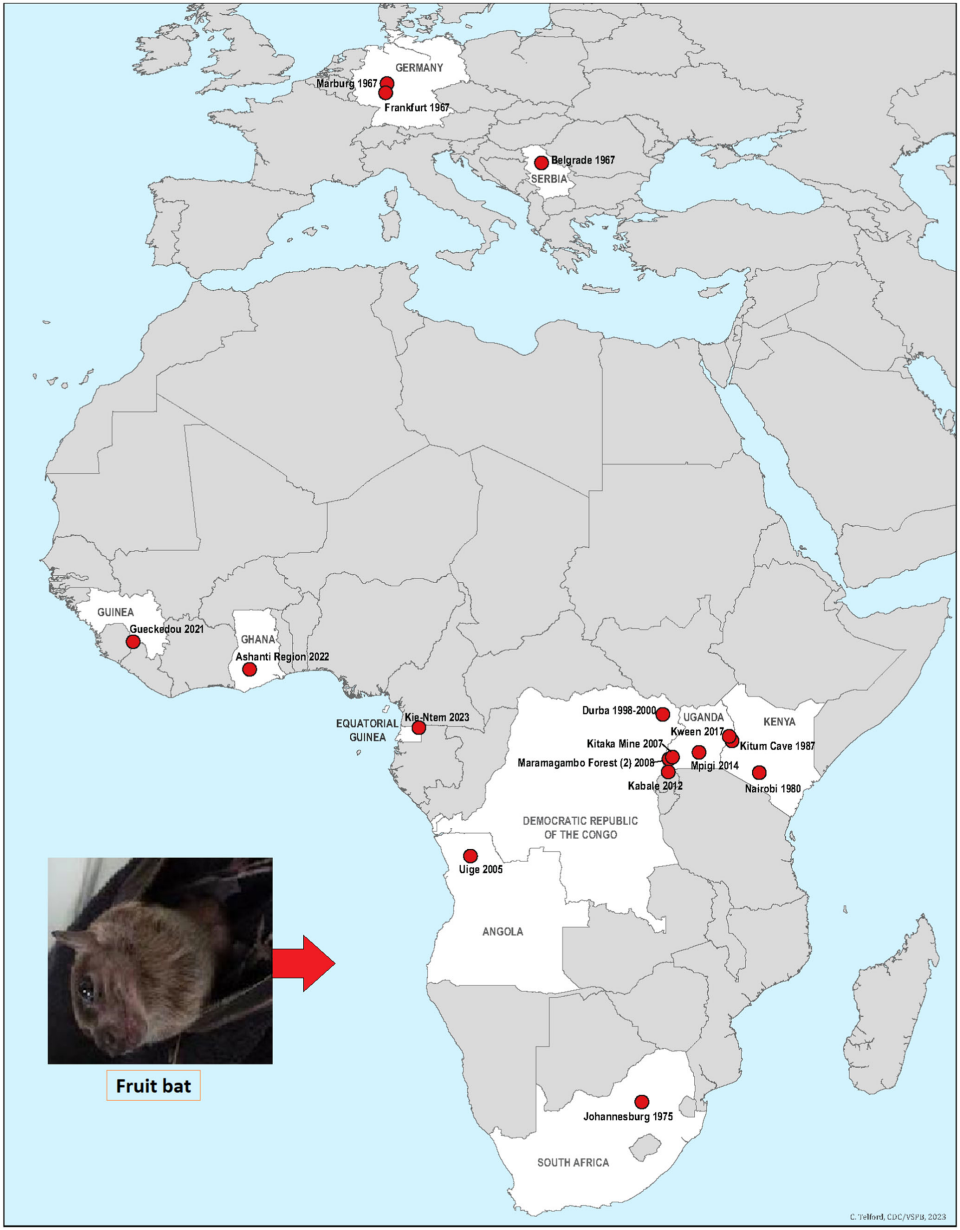
Global reach of the reported MVD outbreaks as by February 23, 2023 (https://www.cdc.gov/vhf/marburg/images/marburg2021.png?_=93408?noicon).

Hemorrhagic fever cases were first detected simultaneously from the Marburg and Frankfurt cities in West Germany, and Belgrade in Yugoslavia (Serbia) laboratories in 1967. Marburg reported 30 confirmed cases and Belgrade reported two. The CDC states that, MARV was first detected with 31 reported cases in August 1967 in Marburg city, West Germany, thus the nomenclature.^15^, ^16^ The transmission was allegedly triggered with the import of infected African green monkey (*Cercopithecus aethiops*) to develop polio vaccine.^13^ Supposedly the first ever discovery of filovirus,^17^ it caused an uncommon but severe hemorrhagic fever (MVD) in both humans and nonhuman primates. MVD cases were reported from Angola, Congo, Kenya, South Africa, and Uganda (Africa) soon after this incident (Figure 1).^1^ Sporadic MVD instances and outbreaks were reported from several countries since then in the late 20th century (Table 1).^18^, ^19^ Nonetheless, most reported cases were from Africa with sporadic reports from Asia, North America, and Europe having an African link.

**Table 1:** Various Marburg virus disease outbreaks reported worldwide.

Year, month	Location	Outbreak linked to	Reported cases	Reported deaths (%)
1967, August	Marburg and Frankfurt, West Germany; Belgrade, Serbia (Yugoslavia)	Handling African green monkeys from Uganda in the laboratory	31	7 (22.6)
1975, February	Johannesburg, South Africa	Male travelling to Zimbabwe	3	1 (33.3)
1980, January	Kenya	Male visited Kitum Cave, Mount Elgon National Park	2	1 (50)
1987, August	Kenya	Boy visited Kitum Cave, Mount Elgon National Park	1	1 (100)
1990	Russia	While handling African monkey in laboratory	1	1 (100)
1998, October to 2000, September	Durba and Watsa, Democratic Republic of Congo	Workers of a gold mine in Durba	154	128 (83.1)
2004, October to 2005, July	Uige Province, northern Angola	Unknown source	374	329 (88)
2007, June to September	Kamwenge and Ibanda Districts, Uganda	Young male workers in Lead and gold mines	4	2 (50)
2008, January	United States	A Uganda‐returned traveller	1	0 (0)
2008, July	The Netherlands	Uganda‐returned woman	1	1 (100)
2012, October	Kabale, Ibanda, Mbarara, Kampala districts, Uganda	Unidentified source	18	9 (50)
2014, September	Kampala District, Uganda	Unidentified source	1	1 (100)
2017, October	Kween District, Eastern Uganda	Dwelling near a bat‐infested cave	3	3 (100)
2021, August	Guéckédou, Republic of Guinea	Unidentified source	1	1 (100)
2022, July	Ashanti region, Republic of Ghana	Unidentified source; genetic sequencing indicated that the genome sequence related to Equatorial Guinea, Sierra Leone and Angola sequences	4	3 (75)
2023, February	Equatorial Guinea	Unidentified origin	40	35 (75)
2023, June	Bukoba, United Republic of Tanzania	Travelled to Goziba island in Lake Victoria, Tanzania	9	6 (66.7)

*Source*: Adopted from the CDC (https://www.cdc.gov/vhf/marburg/outbreaks/chronology.html#six).

The reported MVD cases were only a few before 1998. While three confirmed cases and one death were reported from South Africa in 1975, two cases were from Kenya in 1980 and one in 1987. One confirmed MVD case and related death was reported from Russia in 1990. There were two significant MVD outbreaks in the Democratic Republic of Congo (DRC) and Angola between 1998 and 2000. DRC reported 154 confirmed cases with 83% and Angola reported 422 confirmed MVD cases with 90% fatality rates. The Angola outbreak is the largest ever, with at least 356 deaths out of the 422 confirmed cases occurring only in the Uige province. From the lot, 21 contacts were treated in the Uige municipality and 111 were followed up elsewhere.^20^ Subsequent outbreaks were in Uganda in 2007, 2012, 2014, and 2017, with 27%–100% case fatality rates. The first MVD case in the Republic of Guinea was reported in August 2021 amid the ongoing COVID‐19 pandemic, signaling a new health concern.^19^ Ghana reported four confirmed cases and three associated deaths in 2022.^3^ Table 1 depicts the timeline of the occurrence of MVD. Ugandan grivets (*Chlorocebus aethiops*), their tissues or organs transmitted the virus to laboratory personnel in Germany and Yugoslavia.^13^, ^21^ Contact with the monkey was the primary cause of the usually reported nosocomial transmission.^11^ Numerous deaths with suspected hemorrhagic fever in the Equatorial Guinea were reported by their Ministry of Health and Social Welfare. The real‐time polymerase chain reaction (RT‐PCR) of the samples on February 12, 2023 confirmed one MVD positive case.^22^ A 34‐year‐old male traveler to Central Africa was the first reported suspected case from Valencia, Spain.^23^ More than 200 people in Equatorial Guinea were MVD quarantined, as per the Spanish health agency.

### 3.2 Epidemiology of MVD

Marburg virus is a zoonotic virus of the Ebola virus family that could be contracted from the animal reservoir fruit‐bat (*R. aegyptiacus*), its contaminated oral secretions that contaminate the partially eaten fruit and excreta.^24^, ^25^ It carries the virus without falling sick being the reservoir, however severe‐to‐fatal viral hemorrhagic fever could occur when the virus infects a human. Bat species like *Hipposideros caffer* and other Chiropterans also are likely source of infection.^26^ The virus spreads through direct contact with the wound, scrape, or mucous membranes of the eyes, nose, or mouth. It could also spread through blood or body fluids like urine, sweat, faeces, vomit, breast milk, amniotic fluid, and sperm of an alive or dead MVD patient.^1^ Also, it could be contracted from contaminated inanimate objects especially in hospital settings, like the medical supply, needle, bedding, or clothing in the MVD patient's ward. Another transmission object could be the sperm from oral, vaginal, or anal sexual activity of a MVD survivor, as the virus still persists in bodily fluids (including sperm) even though a convalescing patient may not exhibit acute MVD illness symptoms. MARV transmission by interacting with or touching the vaginal secretions of a MVD patient is not evidenced.

With varying (2–21 days) incubation period,^1^ MVD fatality ranges from 22.6% to 100% (Table 2). MVD manifests as high fever, severe headache, and severe malaise. Abdominal pain, severe watery diarrhoea, nausea, and vomiting may follow from the third day. This shall follow severe hemorrhagic manifestations from fifth to seventh day with the patient bleeding from multiple areas in fatal cases. Due to heavy loss blood loss and shock, death mostly occurs by the eight or nineth day. Clinical diagnosis of MVD is confusing with other tropical febrile illnesses (malaria, typhoid, shigellosis, meningitis, and other VHFs) during early stage due to similar clinical symptoms.^28^ Several MVD diagnostic techniques including reverse transcription‐polymerase chain reaction (RT‐PCR) for viral RNA identification and enzyme‐linked immunosorbent assay (ELISA) for antibody detection are available. Further, MARV infection is confirmed through serum neutralisation test, virus isolation through cell culture, and electron microscopy.^29^ Being extremely biohazardous, sample collection is risky, and thus laboratory testing may be carried out under maximum biological containment setting. All such specimen may be triple‐packaged while transporting. Nevertheless, it necessitates expert laboratory set‐up with skilled manpower that is often lacking in a resource‐limited region where MVD outbreaks are frequent.^30^, ^31^

**Table 2:** Various vaccines developed against MVD worldwide.^27^

Vaccine type	Description
cAd3	Chimpanzee adenovirus serotype 3 vector, encoding glycoprotein of wild‐type Marburg virus
MVA‐BN‐Filo	Modified vaccinia Ankara vector, encoding glycoproteins of Ebola (Sudan), Marburg viruses, and nucleoprotein of Tai Forest virus
DNA plasmid vaccine	Marburg DNA plasmid expressing Marburg (Angola) glycoprotein
rVSV‐MARV‐GP	Recombinant vesicular stomatitis virus vector for Marburg glycoprotein
VLP	Virus‐like particles with glycoprotein

### 3.3 Marv zoonosis

There is a constant threat of the emergence of infections given the crossover potential of microbes from animals and eco‐habitats to humans. Last few decades saw the emergence and reemergence of various microbial diseases of zoonotic origin like the COVID‐19 and mpox.^32^, ^33^, ^34^ The emerging and reemerging of such diseases especially in the African continent is reportedly on the rise.^35^ A major contributing factor to the spread of zoonotic infections is the unhindered crossborder mobility of animals. This is glaringly evident from the emerging dengue and chikungunya cases in the European region that were nonexistent.^36^ The science, social, and natural science research could be integrated for better preparedness and effective management of zoonotic diseases.^37^

The fruit‐bat (*R. aegyptiacus*) is considered the primary reservoir,^1^, ^24^, ^25^ although other Chiropterans like *H. caffer* also are. In view of the fact that MVD is linked to bats, research on bat‐colonizing microflora has been of interest. Bats carry Coronavirus, Paramyxoviridae and Rhabdoviridae among several other viruses. Colonizing microbes may be analyzed using next‐generation sequencing (NGS) and metagenome‐based surveillance to identify prevailing microbial species to predict future outbreaks and implement effective preventive measures.^38^, ^39^

## 4 MITIGATION STRATEGIES

The virus is assumed to zoonotically (animal–human) spread through fruit‐bat, and can spread between humans through contacts with the body fluid, chapped skin, and unprotected intercourse with the infected. The illness has Ebola‐like symptoms of fever and hemorrhage. Although related, Marburg virus is not the same as Ebola virus. The unceasing hysteria around Ebola virus disease contributed significantly to absurd actions by the community during outbreaks.^6^ The MVD seems more severe and the survivor and the family are often stigmatized. MVD epidemic poses significant global public health emergency and mandates global cooperation to tackle it effectively. As the affected country may be resource‐poor to counter the illness, the WHO, the World Bank, the International Monetary Fund (IMF), and others could extend technical and financial aids to garner necessary resources.^40^ Transmission of MVD via sexual route is noticed like the recently reemerged mpox.^21^ Thus, initiating community involvement is important as a measure in the course of preventive strategies.

## 5 CURRENT TREATMENT STRATEGIES

No specific approved antiviral treatment for MVD is yet available, and hydration, pain management, and secondary infection treatment remain the mainstay as supportive care.^41^ Dehydration is managed and organ function is maintained by administering intravenous fluids and replacing electrolyte. Mortality rate remains high due to rapid progression of the disease and limited treatment alternatives. Further, the availability and accessibility of these life‐saving interventions in the outbreak‐prone regions is challenging. Therapeutics (drugs) and prophylactics (vaccines) are at advance research and development stages, offering hope to control future outbreak. For instance, targeted lipid‐encapsulated siRNA inhibited MVD virus replication and reduced the mortality as demonstrated in animal model.^42^

## 6 VACCINES DEVELOPMENT

Vaccines seem to be crucial in current situation when several outbreaks are encountered.^43^ Availability of licensed vaccines/medications and timely treatment of MVD symptoms like dehydration significantly increases the likelihood of survival, the WHO states.^44^ Supportive hospital care to manage patient's fluids, balance the electrolyte, maintain the blood pressure and oxygen levels, restore the lost blood, addressing the clotting components, and treating an exacerbating infection is important. In line with the Ebola response strategy, healthcare system is making all efforts to curb the spread of MVD virus, from raising public awareness, sensitizing, and supporting them to mobilizing the resources. Nonhuman primates have been treated effectively that are yet to be tried on humans.^45^ Merck developed a potential recombinant vesicular stomatitis viral vaccine (rVSV‐MARV) against MVD for Canada Public Health Agency to be discontinued later. Numerous MARV vaccines being researched seem promising. Recombinant VSV‐based MARV glycoprotein expressing vaccine (VSV‐MARV) provided quick protection to animal models against MVD. It is claimed about a candidate vaccine MVA‐BN‐Filo that contains antigens from both Marburg and Ebola viruses to protect against both the hemorrhagic diseases. Its phase 3 trial against Ebola is ongoing and yet to be tested against MARV. The search is still on for efficient prophylactic and therapeutic (like MARV‐specific monoclonal antibody and small‐molecule antivirals) interventions against MVD. A monoclonal antibody (MR186‐YTE) and antiviral remdesivir combination treated nonhuman primate model against MVD successfully.^46^ Chimpanzee adenovirus vectored (cAd3‐Marburg) vaccine was tried on 40 healthy human volunteers and the clinical response titre was estimated. Although it induced neutralizing antibodies in 95% cases, results demonstrated that nearly half of the volunteers developed mild vaccination side‐effects like pain at injection site, malaise, headache, and myalgia.^47^

## 7 THE STATUS OF THE NEGLECTED DISEASE

The WHO classifies MVD as a neglected tropical disease, owing to its neglected research, funding, and attention status as compared to other infectious diseases. Lack of commercial incentives for drug development and limited investments in MVD vaccine development have hindered the progress.^48^ In view of this, the global health agencies, governments, and nongovernmental organizations may prioritize MVD‐specific research and development. Strengthening the surveillance and diagnostic capacities in particularly the endemic regions may aid in expeditious detection and response, and could also potentially prevent an outbreak from ramifying.

## 8 RISKS ASSESSMENT

Making the general public aware about the risk factors and involving the community is an effective way to reduce viral transmission. Considering the greater risk, miners and tourists visiting the fruit‐bat inhabiting caves should follow adequate safety measures. They as also the treating healthcare workers need to be cautious and wear personal protective equipments (PPE), face protection (medical mask and goggles), and gloves as long as it is practicable. Healthcare and laboratory personnel could use nonsterile long‐sleeved gown while dealing with suspected or confirmed MVD cases. Animal products may thoroughly be cooked before consumption to reduce the risk of the zoonotic (bat‐to‐human) transmission. Basic preventative measures in piggeries are warranted as pigs could potentially amplify an outbreak.^29^ The WHO recommends safe sex (semen must test negative) for convalescing male survivors of MVD to prevent possible risk of sexual transmission.

Table 2 details the numerous vaccines developed to counter MVD. Despite the support “One Health” in Ghana, budgeting, coordinating, and executing the cross‐sector initiatives were difficult.^40^ International federations could step up and expedite efforts to support Ghana on this considering the potentially terrible global ramifications of MVD. The spread of MVD must be nipped at the bud before it is too late to avoid the repetition of what was globally encountered with COVID‐19 and Ebola.^49^, ^50^ After confirming the presence of Filovirus antibody in Ghanaian fruit‐bats, research to comprehend the role of the bats as hosts needs revamping. The world has witnessed 17 reported MARV outbreaks so far in Africa with high infectivity and death rate which is a significant public health worry. Thus, tacking and managing MVD appears to be an international issue sooner‐or‐later that calls for global cooperation. Research on anti‐MARV vaccines and antivirals development may be prioritized and expedited.

## 9 THE ONE‐HEALTH APPROACH

The countries in the African continent have prioritized zoonotic illnesses as important public health concern to collaboratively tackle and close the gap through the cooperation of various departments that deal with humans, animals, and the environmental health through the “One Health” strategy. The Ghana Health Service (GHS) has worked to improve its monitoring and laboratory service in similar lines, by creating a national action plan for health security (NAPHS). The action plan trains the scientists, doctors, veterinarians, and paramedics through field epidemiology and laboratory training programe (GFELTP) scheme. This programe is in line with the “One Health” concept to educate the diverse healthcare workers through a 360° approach. Developing the state‐of‐the‐art laboratory diagnosis and surveillance capabilities particularly for the lately identified viruses from filovirus, orthomyxovirus, paramyxovirus, and coronavirus families of major health concerns is necessary. The one health workforce (OHW) project is a part of the USAID focusing on intersectoral disease surveillance, training, and outbreak response. Developing a workforce, monitoring systems for zoonoses, enhancing the national laboratory infrastructure, and prioritizing an action plan for zoonotic illnesses are some aspects that could be promoted so as to improve the capacity to quickly detect and respond to zoonotic infections in line with “One Health”.

## 10 GLOBAL PUBLIC HEALTH IMPLICATIONS

Despite its regional prevalence, the potential of MVD to internationally ramify is a pressing concern. Trade globalization, increased travel, and migration would facilitate global spread of the virus, potentially leading to outbreaks in nonendemic regions. To begin with, addressing the MVD issue requires international collaboration, research funding, and capacity‐building especially in the endemic regions. The “global health security agenda,” an international partnering aiming at preventing, detecting, and responding to threats of infectious diseases, could prioritize MVD for surveillance and preparedness. The MVD could additionally effectively be addressed through coordinated efforts of public health agencies, the policymakers, and the researchers through information and resource sharing.^51^

## 11 CONCLUSION

MVD is one of the neglected infectious diseases that can potentially impact public health at the global scale due to its high (∼90%) fatality rate. Recent outbreaks call for broader and detailed studies on this highly virulent zoonotic disease. A glaring reason for the continuous emergence of the MVD‐zoonotic spill‐over is the chance or deliberate human encounter with the bats in the wild especially near the caves where they primarily dwell. A secondary reason that makes the situation worse is the absence of befitting therapeutic and preventive countermeasures to control MVD disease. Thankfully, large MVD outbreaks are rare although the world has witnessed multiple outbreaks. Also, there is a wide gap on the knowledge of host–virus interactions. Designing evidence‐based recommendations to prevent and control MVD is recommended. Implementing “One Health” strategy to know the host–virus interactions need prioritization. Therefore, the necessity of a high‐level global cross‐discipline joint effort among public health agencies, physicians, paramedics, veterinarians, environmentalists, and policymakers is urgently being felt.

## AUTHOR CONTRIBUTIONS

**Devang Srivastava**: Conceptualization; writing—original draft. **Lakshmi Venkata Simhachalam Kutikuppala**: Writing—original draft. **Pooja Shanker**: Writing—original draft. **Rudra Narayan Sahoo**: Writing—original draft. **Gurudutta Pattnaik**: Writing—original draft. **Rasmita Dash**: Writing—original draft. **Venkataramana Kandi**: Writing—original draft. **Azaj Ansari**: Writing—original draft. **Snehasish Mishra**: Project administration; Writing—review and editing. **Dhruv N. Desai**: Writing—original draft. **Ranjan K. Mohapatra**: Supervision; Writing—review and editing. **Ali A. Rabaan**: Validation; Writing—original draft. **Md. Kudrat‐E‐Zahan**: Writing—original draft.

## CONFLICT OF INTEREST STATEMENT

The authors declare no conflict to declare.

## TRANSPARENCY STATEMENT

The lead author Ranjan K. Mohapatra, Md. Kudrat‐E‐Zahan affirms that this manuscript is an honest, accurate, and transparent account of the study being reported; that no important aspects of the study have been omitted; and that any discrepancies from the study as planned (and, if relevant, registered) have been explained.

## Data Availability

The authors have nothing to report.
